# Cluster correlation based method for lncRNA-disease association prediction

**DOI:** 10.1186/s12859-020-3496-8

**Published:** 2020-05-11

**Authors:** Qianqian Yuan, Xingli Guo, Yang Ren, Xiao Wen, Lin Gao

**Affiliations:** School of Computer Science and Technology, XIDIAN UNIVERSITY, Xi’an, Shaanxi China

**Keywords:** Long noncoding RNA, Disease, lncRNA-disease association, Cluster correlation, Bipartite network

## Abstract

****Background**:**

In recent years, increasing evidences have indicated that long non-coding RNAs (lncRNAs) are deeply involved in a wide range of human biological pathways. The mutations and disorders of lncRNAs are closely associated with many human diseases. Therefore, it is of great importance to predict potential associations between lncRNAs and complex diseases for the diagnosis and cure of complex diseases. However, the functional mechanisms of the majority of lncRNAs are still remain unclear. As a result, it remains a great challenge to predict potential associations between lncRNAs and diseases.

**Results:**

Here, we proposed a new method to predict potential lncRNA-disease associations. First, we constructed a bipartite network based on known associations between diseases and lncRNAs/protein coding genes. Then the cluster association scores were calculated to evaluate the strength of the inner relationships between disease clusters and gene clusters. Finally, the gene-disease association scores are defined based on disease-gene cluster association scores and used to measure the strength for potential gene-disease associations.

**Conclusions:**

Leave-One Out Cross Validation (LOOCV) and 5-fold cross validation tests were implemented to evaluate the performance of our method. As a result, our method achieved reliable performance in the LOOCV (AUCs of 0.8169 and 0.8410 based on Yang’s dataset and Lnc2cancer 2.0 database, respectively), and 5-fold cross validation (AUCs of 0.7573 and 0.8198 based on Yang’s dataset and Lnc2cancer 2.0 database, respectively), which were significantly higher than the other three comparative methods. Furthermore, our method is simple and efficient. Only the known gene-disease associations are exploited in a graph manner and further new gene-disease associations can be easily incorporated in our model. The results for melanoma and ovarian cancer have been verified by other researches. The case studies indicated that our method can provide informative clues for further investigation.

## Background

About 3% of the human genome is the coding region, which produces multiple proteins, and other non-coding regions transcribe a large number of non-coding RNAs. Much of the non-coding region of the human genome has historically been regarded as junk DNA [1]. However, for decades, researchers have discovered that multiple types of RNA exist, and among the most important is non-coding RNA (ncRNA). According to transcript lengths, ncRNAs could be further categorized into small ncRNAs and lncRNAs [2]. LncRNAs are the biggest part of non-coding RNAs which are longer than 200 nucleotides and are not translated into proteins [3, 4]. It is estimated that about 62% of the human genome is transcribed to produce long non-coding RNAs. Compared with protein-coding transcripts, lncRNAs have fewer exons and are expressed at lower levels [5, 6]. However, lncRNAs show extensive mechanisms to play their biological roles compared to small ncRNAs [7]. As shown by more and more studies that lncRNAs play crucial functional roles in cytoplasm and nucleus through cis or trans-regulatory mechanisms [6], and play important roles in different cellular pathways [8, 9].

In recent years, with the rapid development of high-throughput sequencing technologies, researchers have identified many lncRNAs in eukaryotic organisms. For example, Cabili et al. integrated chromatin marks and RNA-sequencing data to identify more than 8000 long intergenic ncRNAs across 24 different human cell types and tissues [10]. And accumulating evidences have shown that mutations and disorders of lncRNAs are closely related to many complex human diseases [11]. The earliest lncRNAs to be discovered were XIST [12] and H19 [13]. These two genes have been demonstrated to be linked to several types of cancers. For example, One of the first lncRNAs to be identified, H19, acts as a decoy for several tumor suppressor miRNAs, with let-7 [14]. Another important discovery of lncRNAs is that the lincRNA termed HOTAIR is increased in expression in primary breast tumors and metastases, and HOTAIR expression level in primary tumors is a powerful predictor of eventual metastasis and death [15]. Yan et al. comprehensively analyzed the characteristics of lncRNAs in different types of human cancers at the genome, transcription and epigenetic levels [16]. The results indicated that lncRNAs are more specific than mRNAs in expression and dysregulation in different cancers [16]. With regard to liver cancer, Yang et al. not only analyzed the dysregulated lncRNAs, but also inferred its pathogenesis by combining methylation and copy number variation [17]. Due to their functional significance, various databases have been developed to store lncRNA related information, such as lncRNAdb [18], NONCODE [5], including the information of lncRNA structure, expression, and so on. LncRNADisease [19], Lnc2Cancer [20] are mainly focused on different lncRNA-disease associations. These databases are crucial for deciphering lncRNA functions in human diseases. However, the functions and biological relevance of the vast majority of lncRNAs remain enigmatic.

Recently, the functions of lncRNAs and their associations with human diseases have attracted much attention from researchers because increasing evidences indicated that lncRNAs play critical roles in the development of various human diseases. With the development of novel experimental and computational methods, researchers have proposed a variety of models to predict the biological functions of lncRNAs and lncRNA-disease associations. For example, Chen et al. constructed a computational tool named LRLSLDA to predict novel human lncRNA-disease associations [21]. It is well known that LRLSLDA is the first lncRNA-disease association prediction model which is based on the assumption that the functions of lncRNAs associated with similar diseases are often similar. A semi-supervised learning framework of Laplacian Regularized Least Squares was mainly applied in this model. As a result, LRLSLDA significantly improved the performance of previous methods used to solve the similar computational biology problems. Based on the basic assumption that similar diseases tend to have associations with functionally similar lncRNAs, more computational models were developed, such as LNCSIM [22] and LDAP [23]. LNCSIM calculated lncRNA functional similarity on a large scale based on lncRNA-disease associations and disease semantic similarity. LDAP was proposed to predict potential lncRNA-disease associations by using a bagging SVM classifier based on lncRNA similarity and disease similarity. Furthermore, some models were developed by integrating multiple data sources into networks. In 2015, Guo et al. developed a reliable method named lncGFP [24] based on a global network strategy to predict probable functions of lncRNAs at large scales, which may give clues to the potential associations between lncRNAs and diseases. Sun et al. proposed a computational method named RWRlncD [25] by implementing random walk with restart (RWR) on the lncRNA functional similarity network. Chen et al. developed model named IRWRLDA [26] which combined lncRNA expression similarity and disease semantic similarity to set the initial probability vector of the RWR to predict novel lncRNA-disease associations. Yang et al. constructed a coding-non-coding gene-disease bipartite network based on the known gene-disease associations and uncovered the hidden lncRNA-disease associations by implementing a global propagation algorithm on this network [27]. Chen et al. developed a model called KATZLDA by integrating known lncRNA-disease associations, lncRNA expression profiles, lncRNA functional similarity, disease semantic similarity and Gaussian interaction profile kernel similarity to uncover potential lncRNA-disease associations [28]. Furthermore, KATZLDA could work for both new diseases and lncRNAs. Due to few known lncRNA-disease associations, some researchers have developed methods that rely on other information besides the known lncRNA-disease associations. For example, Liu et al. identified potential lncRNA-disease associations based on known gene-disease associations and gene-lncRNA co-expression relationships which was the first computational method without the need to rely on known lncRNA-disease associations [29]. All the lncRNA-disease association prediction models aforementioned were listed in the Table S1(see Additional file 1).

In this paper, a simple and efficient method was developed to predict novel lncRNA-disease associations. First, a bipartite network is constructed by integrating known lncRNA-disease associations and protein-coding gene-disease associations. Then the concept of disease clusters and gene clusters is presented in the bipartite network. The key idea behind this method is that the nodes in one part associated with the same node in another part are more similar with each other, which is similar to the assumption used by other methods [22, 23, 26]. Based on the above, we proposed a new method to calculate association scores for potential gene-disease pairs. Cross-Validation tests were used to evaluate the performance of our method. As a result, our method obtained reliable AUCs of 0.8169, 0.8410 in the LOOCV based on Yang’s [27] dataset and Lnc2Cancer 2.0 [30] database, respectively. We further implemented 5-fold cross validation on our method and obtained reliable AUCs of 0.7573, 0.8198 based on Yang’s dataset and Lnc2Cancer 2.0 database, respectively. The performance of our method was superior to other similar methods on the two datasets. Moreover, case studies on melanoma and colon cancer demonstrated that it could give clues to further investigations.

## Results

### Prediction of lncRNAs associated with diseases

For the gene-disease pairs without edges in the bipartite network, our method can calculate an association score for a pair which can be used to measure the potential association strength of this gene-disease pair. Ultimately, we sorted the association scores of all potential gene-disease pairs and selected the top 1% as predicted results, and obtained a total of 2320 potential gene-disease associations (1321 lncRNA-disease pairs and 999 protein coding gene-disease pairs) (see Additional file 2).

### Performance evaluation

LOOCV and 5-fold cross validation were applied to evaluate the prediction performance of our method based on known lncRNA-disease associations from the dataset of Yang [27] and Lnc2Cancer 2.0 database [30]. When LOOCV was applied, each known lncRNA-disease association was removed from the lncRNA-disease bipartite network in turn as test sample. Our method was assessed by how well the removed lncRNA-disease association was ranked within all the lncRNA-disease associations. The receiver operating characteristics (ROC) curve can be obtained by plotting true positive rate (TPR) versus false positive rate (FPR) at different rank thresholds. Given the rank threshold k, TPR indicates the percentage of the removed edges with ranks higher than the threshold and FPR indicates the percentage of negative samples with ranks higher than this threshold. Therefore, ROC can be drawn and area under ROC curve (AUC) could be further calculated (see Additional file 3). Considering the isolated nodes whose unique edges were removed, we cannot obtain any relevant information about them. So we removed all the nodes whose degrees were one before we performed LOOCV. The dataset of Yang contained 236 lncRNA-disease associations between 102 diseases and 44 lncRNAs (see Additional file 4). And 1541 lncRNA-disease associations between 249 lncRNAs and 85 diseases were obtained from the Lnc2Cancer 2.0 database (see Additional file 4).

**Fig. 1:**
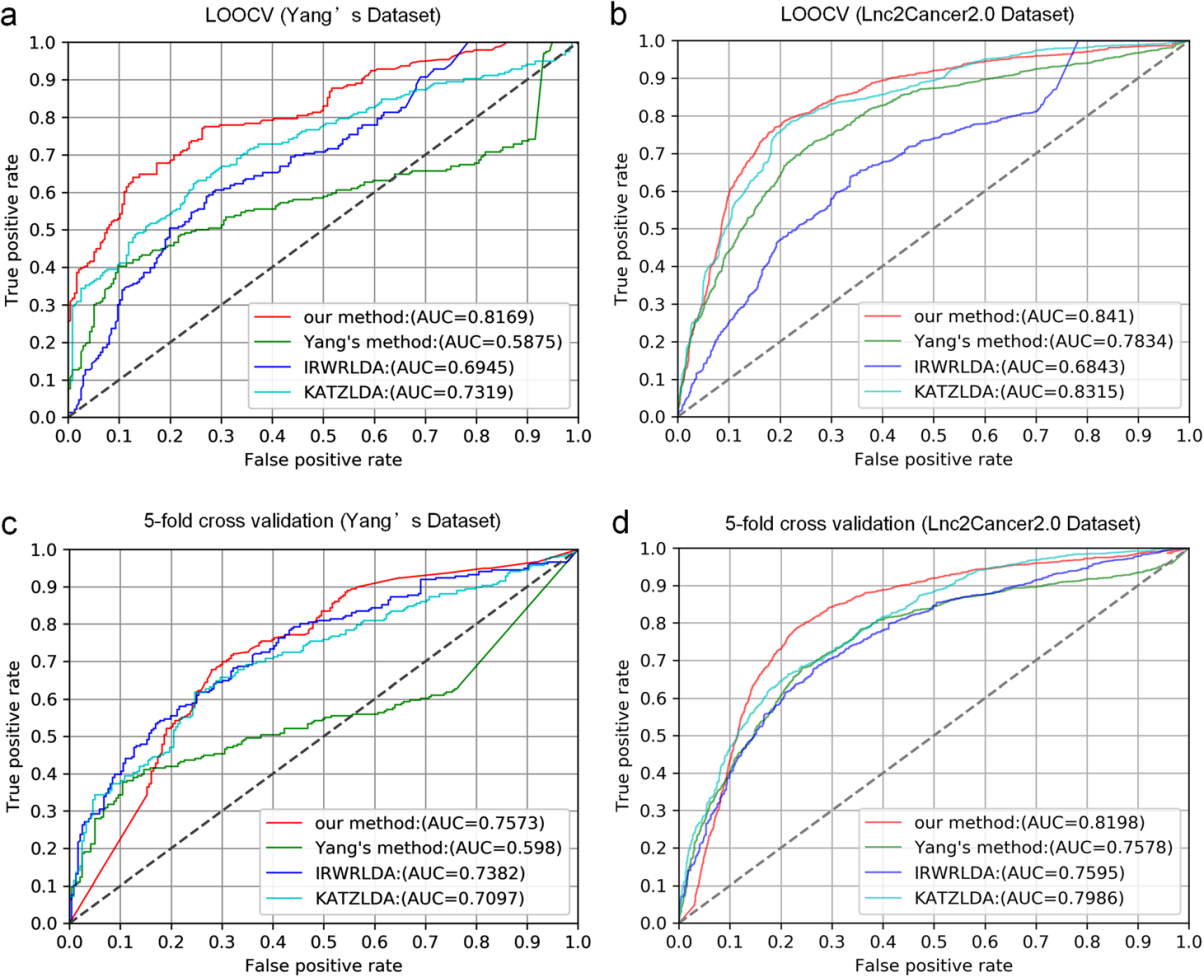
Cross validation tests of our method. **a** Comparative results of LOOCV on Yang’s dataset. **b** Comparative results of LOOCV on the Lnc2Cancer 2.0 dataset. **c** Comparative results of 5-fold cross validation on Yang’s dataset. **d** Comparative results of 5-fold cross validation on the Lnc2Cancer 2.0 dataset

Our method was compared with the following three state-of-the-art methods (Yang’s method [27], IRWRLDA [26] and KATZLDA [28]) by cross validation tests on two datasets (Yang’s dataset and Lnc2Cancer 2.0). In LOOCV tests and 5-fold cross validation tests, the performance of our method was superior to other three methods. Details for LOOCV tests can be seen in Fig. 1a and b, and the results of 5-fold cross-validation tests were shown in Fig. 1c and d.

### Robustness of our method

**Fig. 2:**
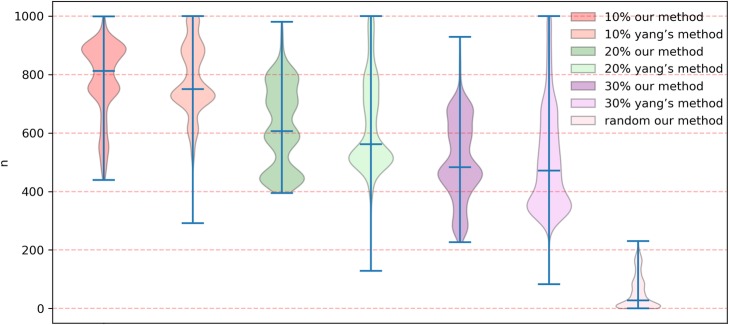
Comparative results of robustness test between our method and Yang’s method at different thresholds. The vertical ordinate indicated the times predicted correctly in 1000 re-sampling experiments between our method and Yang’s method at each different threshold. And the last graph represents times predicted correctly in 1000 re-sampling experiments on the random network by our method

To test the robustness of our method in a network view for predicting potential gene-disease associations, the method of Multiple Survival Screening (MSS) [31] is used to test our method under perturbation of the bipartite network. First, a total of 2320 potential gene-disease associations in our bipartite network was used to evaluate the performance of our method in these perturbation tests, which is called the set of verification edges. Then, a certain percentage of edges (10, 20, 30%, respectively) in the network were deleted randomly in these tests. Our method was utilized to predict potential associations on these remaining networks. The performance is evaluated on the verification set. At each different threshold, re-sampling experiments are performed 1000 times. A vector of size 2320 was constructed, corresponding to 2320 predicted edges. Each value in the vector represented the times of its corresponding verification edge could be predicted in 1000 experiments. Our method was more stable in comparison with Yang’s method at different thresholds (Fig. 2). The results of 1000 re-sampling experiments at different thresholds were shown in Additional file 5. When 20% of edges were deleted, the prediction accuracy could be maintained at around 0.8 which was significantly higher than Yang’s method (*p*-value = 0.022). As the proportion of deleted edges increased, the accuracy decreased gradually (Fig. 2). Here, we also randomly rewired the edges to construct random network, while keeping the degree of each node in the bipartite network unchanged. Our method was also applied to the random network for comparison. The results indicated that the accuracy of our method was significantly higher than that of random network (*p*-value< 10^− 10^).

### Case study

In order to further demonstrate the performance of our method in predicting potential lncRNA-disease associations, the results of colon cancer and melanoma were analyzed as case study. For each case, the genes associated with the disease were ranked according to their association scores. Based on our results (2320 potential gene-disease associations), we retained genes within top 5% related to these two diseases independently for further analysis. Our predictions were validated by other independent experiments, part of which were listed in Table 1.

**Table 1:** Case studies of colorectal cancer and melanoma

LNCRNA	Disease	PMID	Rank
MIR31HG	Colorectal cancer	30,195,788	Top23
CCND1	Colorectal cancer	27,191,497	Top23
lncRNA-HEIH	Colorectal cancer	29,081,216	Top28
LSINCT5	Colorectal cancer	25,526,476	Top29
MIR31HG	Melanoma	25,908,244	Top32
U47924.27	Melanoma	28,225,791	Top32
CCND1	Melanoma	23,001,925	Top32

Colorectal cancer (CRC) is a common malignant tumor of the digestive tract that occurs in the colon. In recent years, the prevalence rate of colorectal cancer has increased continuously [32]. The studies indicated that lncRNAs played an important role in the development and progression of colorectal cancer [33]. There were 31 lncRNAs predicted to have potential associations with colorectal cancer by our method. Part of them were validated by other independent experiments. For example, Zhou et al. determined that MIR31HG was closely related to the recurrence of colorectal cancer [34]. The signature of MIR31HG held great potential for risk assessment of recurrence and personalized management of colorectal cancer patients. Chen et al. observed that miR-374a inhibited colorectal cancer progression by reducing CCND1 to inactivate the PI3K/AKT pathway [35]. Cui et al. demonstrated that lncRNA-HEIH promoted CRC tumorigenesis through counteracting miR-939–mediated transcriptional repression of Bcl-xL, and suggested that lncRNA-HEIH may serve as a prognostic biomarker and therapeutic target for CRC [36]. They found that lncRNA-HEIH was significantly increased in colorectal cancer tissues and cell lines. The expression of lncRNA-HEIH was positively associated with tumor size, invasion depth, and poor prognosis of CRC patients [36]. Moreover, Xu et al. found that the expression level of LSINCT5 was closely related to the disease-free survival and disease-specific survival rates based on Kaplan-Meier analysis in CRC patients [37].

Melanoma, also known as malignant melanoma, is a type of malignant tumor derived from melanocytes. As one of the most malignant tumors in skin tumors, melanoma is prone to distant metastasis, so early diagnosis and treatment are particularly imperative. Accumulating evidences have revealed that lncRNAs played critical roles in the development and progression of melanoma. There were 32 lncRNAs predicted to have potential associations with melanoma among our results. Some results were validated by other studies. For example, Montes et al. found that patients with higher levels of MIR31HG often have reduced p16INK4A expression, which suggested that MIR31HG with repression of p16INK4A in these patients favored cancer development [38]. Wang et al. observed that the low expression of U47924.27 was significantly associated with decreased survival in melanoma patients, revealing the potential role of U47924.27 in melanoma tumorigenesis and metastasis [39]. Furthermore, Vízkeleti et al. observed that CCND1 alterations were linked to melanoma progression and CCND1 amplification may have a prognostic relevance in cutaneous melanoma and emphasized that changes in CCND1 gene expression may influence the metastatic progression, survival and metastasis localization [40].

## Discussion

LncRNAs are involved in the regulation of various processes in cells and the development of complex diseases through a variety of biological mechanisms. Therefore, predicting and discovering lncRNAs associated with complex diseases are important for the diagnosis and treatment of diseases. In this paper, we constructed a bipartite network using known gene-disease associations. Then we predicted potential lncRNA-disease associations only based on the topological information of the gene-disease bipartite network. It is assumed that genes (diseases) associated with the same disease (gene) are more similar. The assumption was incorporated into our bipartite network to proposed the definitions of gene clusters and disease clusters. The biological significance of the two kinds of clusters are analyzed in comparison with those in random networks. And then, the problem of predicting potential lncRNA-disease associations was formulated as a problem of measuring the association strength between gene clusters and disease clusters. The ‘ *C* _ *score* ’ index was first defined to estimate the association strength between clusters. Then the gene-disease association score was defined based on the *C* _ *score* with regard to the influence of different degrees of the node in the bipartite network. Cross validation test was applied to evaluate the prediction performance of our method. In comparison with the state-of-the-art prediction methods, our method can achieve better performance in terms of AUC values and robustness. Moreover, case studies of melanoma and colon cancer were implemented to further demonstrate that it could be a useful and simple method for predicting potential relationships between lncRNAs and diseases as well.

However, there are also some limitations existing in our current method. In spite of the fact that our method is significantly superior to the previous methods, its performance can also be improved by incorporating other information in our model. Due to the fact that only the known gene-disease associations were exploited in the model, our method cannot be applied to the diseases without any known associated genes. Further data integration will be helpful to improve the power of our model and characterize the complex relationships between new genes (without any known associated diseases) and new diseases (without any known associated lncRNAs) from different perspectives. For example, the Single Nucleotide Polymorphism (SNP) information, disease similarity information and lncRNA similarity information can be integrated in the network, which will be our further study. Moreover, the advancement of useful models in other fields such as miRNA-disease association prediction [41, 42], drug-target interaction prediction [43] and synergistic drug combination prediction [44], would greatly facilitate the development of lncRNA-disease association prediction.

## Conclusion

In this study, we proposed an effective method for predicting potential lncRNA-disease associations based on a bipartite network. Firstly, the gene-disease bipartite network was constructed based on known gene-disease associations. Then a formula of gene-disease association score was proposed to evaluate the strength of the potential associations between diseases and lncRNAs. Our method was estimated comprehensively by cross-validation, robustness analysis and case studies in comparison with other methods. The results showed that our method had higher prediction accuracy and robustness even if it was simple and easy.

## Methods

### Data sources

The dataset of known gene-disease associations used in this article were from the work of Yang [27], including lncRNA-disease associations and protein-coding gene-disease associations. The lncRNA-disease associations contained two parts. One was from the LncRNADisease [19] database included 480 experimentally confirmed associations between 118 lncRNAs and 166 diseases. The other part was from literature mining included 380 lncRNA-disease associations between 226 lncRNAs and 145 diseases. There were 578 associations between 295 lncRNAs and 214 diseases totally. Besides, protein-coding gene-disease associations from Yang’s study [27] were also incorporated into the current study. Finally, a total of 1558 gene-disease associations between 1096 genes (295 lncRNAs and 801 protein-coding genes) and 214 diseases were merged together to construct the gene-disease bipartite network (see Additional file 6).

Based on the known associations between lncRNAs and diseases, we constructed a bipartite network defined as *G*(*X*, *Y*, *L*). The *X* denoted a set of lncRNA nodes. The *Y* denoted a set of disease nodes in which the nodes were associated with the lncRNAs in *X*. *L* represented a set of edges between the nodes in *X* and the nodes in *Y*. Regarding to the associations between protein-coding genes and diseases which can provide more informative clues, these associations were further integrated into the bipartite network. Thus, the *X* was a family of protein-coding genes and lncRNAs. Ultimately, a bipartite network based on known protein-coding gene/lncRNA disease associations was constructed for the prediction of potential gene-disease associations.

### Disease cluster and gene cluster

**Fig. 3:**
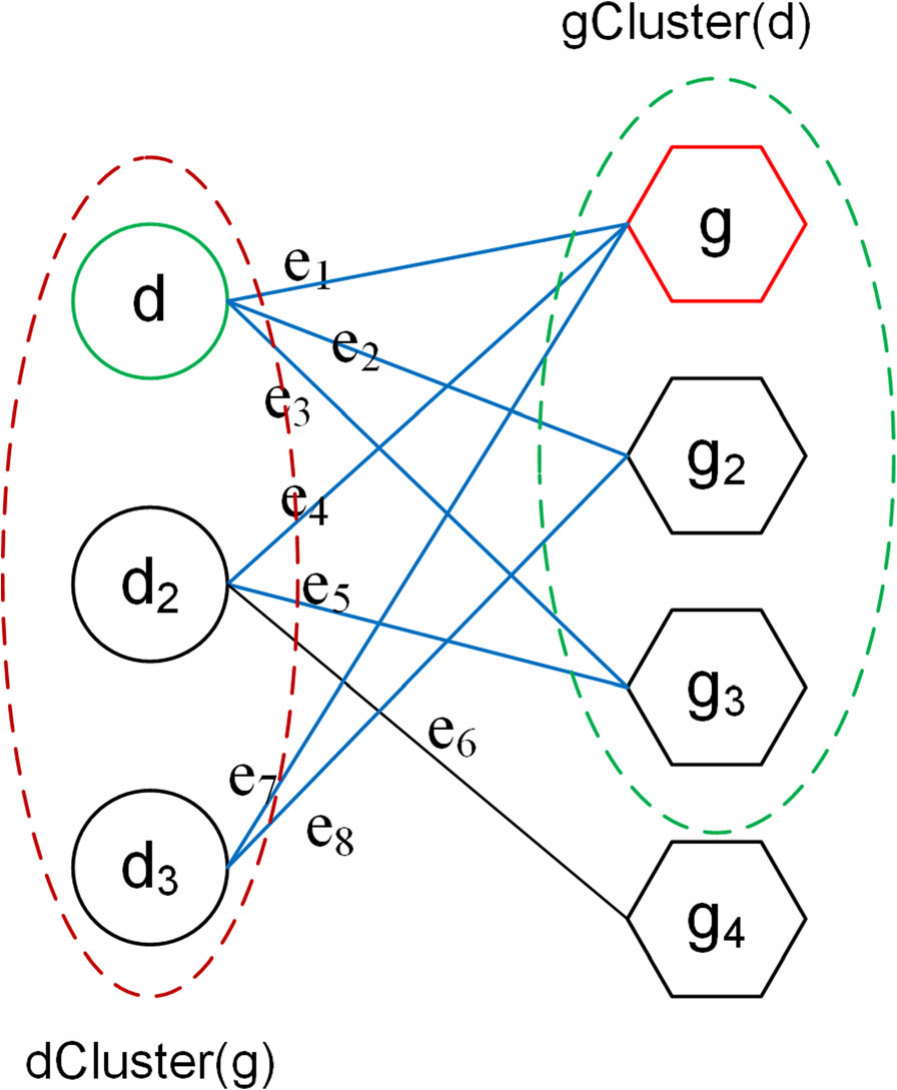
Disease cluster and gene cluster in bipartite network: The circle represented disease node, the hexagon represented gene node, the disease cluster *dCluster*(*g*) of gene *g* was identified by a red dotted line, the gene cluster *gCluster*(*d*) of disease *d* was identified by a green dotted line, and the blue line represented edge between *gCluster*(*d*) and *dCluster*(*g*)

It is assumed that diseases (genes) associated with the mutual genes (diseases) are more similar [45] which was exploited to predict novel gene-disease associations in our work. Therefore, as is for the gene-disease bipartite network *G*(*X*, *Y*, *L*), we defined the notion of disease cluster and gene cluster based on this assumption. For any given disease, we called the collection of its associated genes in the bipartite network as a gene cluster. Similarly, for any given gene, we called the collection of its associated diseases in the bipartite network as a disease cluster. As shown in Fig. 3, the gene cluster of disease *d* was denoted by *gCluster*(*d*) indicated by a green dashed line. The disease cluster of gene *g* was denoted by *dCluster*(*g*) indicated by a red dashed line. Moreover, for any node *v* in the network we built, the *N*(*v*) described a set of nodes linked to *v*. Obviously, we had *N*(*d*) = *gCluster*(*d*) and *N*(*g*) = *dCluster*(*g*).

**Fig. 4:**
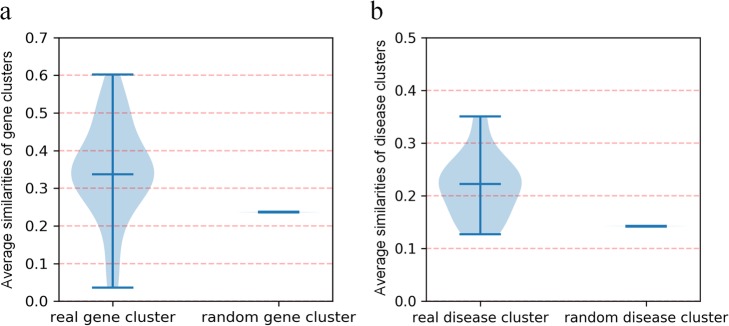
Cluster similarities in the bipartite network. **a** Comparison of functional similarities between real gene clusters and random gene clusters. **b** Comparison of similarities between real disease clusters and random disease clusters

The node similarity in the same cluster was calculated to explore the biological significance of these two kinds of clusters in the bipartite network to facilitate the application of the clusters. For this purpose, we examine the node similarity of these two kinds of clusters first in our bipartite network as follows. For any given gene *g* in the bipartite network, the similarity of corresponding *dCluster*(*g*) was calculated by the average similarity between any two diseases in the cluster. Analogously, the similarity of *gCluster*(*d*) for disease *d* was computed through the average functional similarities of any two genes in the cluster. Next, we constructed different random clusters which had the same size as the corresponding gene/disease clusters. The similarities of random clusters were calculated in the same way (see Additional files 3, 7 and 8). The result of comparative functional similarities of random gene clusters with that of real gene clusters was represented in Fig. 4a. The result of comparative similarities of random disease clusters with that of real disease clusters was represented in Fig. 4b. As expected, the node similarities of real disease clusters were significantly higher than those of random disease clusters in the bipartite network (*p*-value = 0.0004). It can be seen that the comparison of real gene clusters with random gene clusters had comparable results (*p*-value = 0.0003). These results indicated that disease clusters and gene clusters really existed in our bipartite network and may have some biological significance. It is reasonable to infer that the existence of such clusters was due to the fact that nodes in the identical cluster were connected to at least one mutual node in the network. Additionally, the shortest topological distance between any two nodes in a disease cluster or a gene cluster was two, which was the minimum distance between nodes from the same side in a bipartite network. Consequently, for a potential gene-disease pair (*g*, *d*) whose relationship was remain unknown in the bipartite network, we explored the similarity of the disease cluster *dCluster*(*g*) and the functional similarity of the gene cluster *gCluster*(*d*) to calculate the association strength of a potential gene-disease pair (*g*, *d*).

### Calculation of cluster association score

Given a pair of gene-disease association (*g*, *d*), *g* and *d* represented a gene and a disease in the bipartite network, respectively. The cluster association score of the gene cluster corresponding to *d* and the disease cluster corresponding to *g* can be mathematically defined as follows:

1 $$ C\_ score\left(g,d\right)=\left|L\Big( dCluster(g), gCluster(d)\Big)\right| $$

Where *dCluster*(*g*) and *gCluster*(*d*) were disease cluster of gene *g* and gene cluster of disease *d*, respectively. *L*(*dCluster*(*g*), *gCluster*(*d*)) was the edges set in which the element represented the edge between nodes in *dCluster*(*g*) and that in *gCluster*(*d*). In addition, |•| denoted the size of the edges set. The eq. (1) described the cluster association score of a gene-disease pair (*g*, *d*) is used to characterize how heavily the gene cluster was associated with the disease cluster. It was determined by the number of edges between the two clusters. For example, the value of *C* _ *score*(*g*, *d*) in Fig. 3 was 7, because there were 7 connected edges between *dCluster*(*g*) and *gCluster*(*d*) which were drawn by blue lines.

To better verify the performance of *C* _ *score* in measuring the correlations between genes and diseases, we calculated *C* _ *score* values of any gene-disease pairs as long as there was at least one edge between them in the bipartite network. Furthermore, according to the gene cluster and the disease cluster of each edge, we constructed random gene cluster and random disease cluster with the same size, respectively. Then we calculated *C* _ *score* value based on the random gene cluster and the random disease cluster for comparison (see Additional files 3,9). It was interesting that the results indicated that the *C* _ *score* values of real clusters corresponding to the known edges were much greater than that derived from random clusters (Additional file 1, Fig. S1). It can be expected that the *C* _ *score* can provide informative insights into the uncovering the potential disease-gene associations. As a result, the gene-disease association score was defined based on the *C* _ *score* in the following section.

### Calculation of gene**-**disease association score

**Fig. 5:**
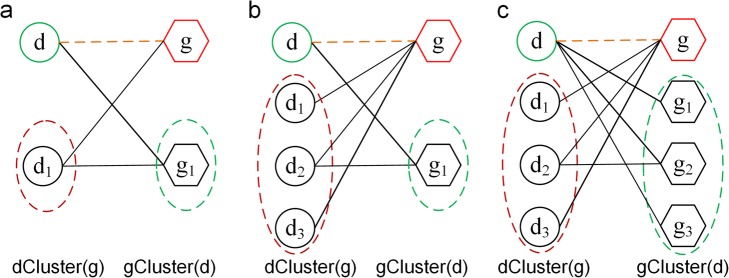
The influence of node degree on the calculation of gene-disease association scores in the bipartite networks. The circle represented disease node, the hexagon represented gene node, the disease cluster *dCluster*(*g*) of gene *g* was identified by a red dotted line, the gene cluster *gCluster*(*d*) of disease *d* was identified by a green dotted line

While there was no known edge between gene *g* and disease *d* in the bipartite network, we can calculate the gene-disease association score based on the aforementioned formula of *C* _ *score* (cluster association score) for gene-disease pair (*g*, *d*). Notably, the value of *C* _ *score* was determined only by the number of connections between two clusters corresponding to the disease and the gene. It was influenced by the degrees of gene *g* and disease *d* in three different types of cases, which was exampled in Fig. 5. It was reasonable to expect that these three distinct types between two clusters may appear in the network, and all of them had a *C* _ *score* value of 1. However, the association strength of the gene *g* and the disease *d* in the three conditions were obviously different. Apparently, the disease cluster corresponding to gene *g* and the gene cluster corresponding to disease *d* in Fig. 5a had the strongest association among three cases. Since the nodes in Fig. 5b and Fig. 5c had higher degrees, their values of *C* _ *score* were equivalent to that in Fig. 5a. It was obvious that the cluster association score was a favorable method for nodes with large degrees. Therefore, considering the influence of nodes degree in the bipartite network, the association score between gene *g* and disease *d* was defined based on the *C* _ *score* and the node degree as follows.

2 $$ DG\_ score\left(g,d\right)=\left(\frac{1}{\left|N(g)\right|}+\frac{1}{\left|N(d)\right|}\right)\times C\_ score\left(g,d\right) $$

Here, *N*(*g*) and *N*(*d*) represented the degrees of gene *g* and disease *d* in the bipartite network, respectively. *C* _ *score*(*g*, *d*) was the cluster association score which can be calculated by the formula (1). The value of *DG* _ *score*(*g*, *d*) reflected the association strength of gene-disease pairs with no known edges in the bipartite network.

## Supplementary information

**Additional file 1.** In this file we provide the supplementary table and figure referred to in the main text.

**Additional file 2.** In this file we provide the results of prediction.

**Additional file 3.** In this file we provide the details about how to calculate the similarity of gene cluster, the similarity of diseases cluster, the gene-disease association score as well as the procedure of leave-one-out cross validation.

**Additional file 4.** In this file we provide the datasets used for cross-validation. In sheet 1 of this file the 236 lncRNA-disease associations from the work of Yang are given. In sheet 2 of this file the 1541 lncRNA-disease associations from the Lnc2Cancer 2.0 database are given.

**Additional file 5.** In this file we provide the result of 1000 resampling experiments at different thresholds.

**Additional file 6.** In this file we provide the information of raw data. In sheet 1 of this file the serial numbers of genes and diseases are given. In sheet 2 of this file the edges in the bipartite network are given. In sheet 3 of this file the concrete information about the abbreviations of diseases is provided. In sheet 4 of this file the concrete information about the abbreviations of genes is provided.

**Additional file 7.** In this file we provide the result of similarities of disease clusters and random disease clusters. In sheet 1 of this file the information of diseases with ID is listed. In sheet 2 of this file the results of disease clusters corresponding to each gene are shown. In sheet 3 of this file the similarity of disease clusters corresponding to each gene and the average similarity of 10,000 random disease clusters with same size are provided. In sheet 4 of this file the average similarity of disease clusters and the average similarity of random disease clusters for each size are given.

**Additional file 8.** In this file we provide the result of functional similarity of gene clusters and random gene clusters. In sheet 1 of this file the information of genes with ID is listed. In sheet 2 of this file the result of gene clusters corresponding to each disease is given. In sheet 3 of this file the similarity of gene clusters corresponding to each disease and the average similarity of 10,000 random gene clusters with same size are shown. In sheet 4 of this file the average similarity of gene clusters and the average similarity of random gene clusters for each size are provided.

**Additional file 9. **In this file we provide the result of *C* _ *score* values. For each edge in the bipartite network, we provide the disease cluster and gene cluster corresponding to the edge as well as its *C* _ *score* value. In addition, we provide *C* _ *score* value of random gene-disease cluster corresponding to each edge which was calculated by 1000 random experiments.

## Data Availability

The datasets supporting the conclusions of this article are included within the article and its additional files.

## Abbreviations

lncRNAs: Long non-coding RNAs
LOOCV: Leave-one out cross validation
ncRNA: Non-coding RNA
RWR: Random walk with restart
ROC: Receiver operating characteristic
TPR: True positive rates
FPR: False positive rates
AUC: Areas under ROC curve
MSS: Multiple survival screening
CRC: Colorectal cancer
SNP: Single nucleotide polymorphism
