# Supplementary material for: Cluster correlation based method for lncRNA-disease association prediction
Source: BMC Bioinformatics. 2020 May 11;21:180. doi: 10.1186/s12859-020-3496-8 (PMC7216352; doi:10.1186/s12859-020-3496-8)
Supplement: Supplementary file 1 — Additional file 1. In this file we provide the supplementary table and figure referred to in the main text. [file 12859_2020_3496_MOESM1_ESM.pdf]

Table S1. Summary of some valuable lncRNA-disease association prediction models.

NAME (DATE)	PUBMED ID	METHOD	DATASETS	CASE STUDIES
LRLSLDA (2013)	24002109	Laplacian Regularized Least Square in the semi-supervised learning framework	disease-lncRNA associations, lncRNA expression profiles	Alzheimer's disease<br>bladder cancer<br>melanoma<br>breast cancer
LNCSIM (2015)	26061969	Laplacian Regularized Least Square, Similarity scores	lncRNA-disease associations, disease semantic similarity	colorectal cancer<br>lung cancer
LDAP (2017)	28172495	Bagging SVM classifier	lncRNA-disease associations, PPI, lncRNA sequence, GO	breast cancer
RWRlncD (2014)	24850297	Random walk With restart on an lncRNA–lncRNA functional similarity network	lncRNA–disease association, disease similarity, gene functional similarity	prostate cancer<br>Alzheimer's disease
IRWRLDA (2015)	27517318	Random Walk with Restart on a heterogeneous network	lncRNA-disease associations, lncRNA expression profiles, lncRNA functional similarity, disease semantic similarity, lncRNA Gaussian interaction profile kernel similarity	colon cancer<br>leukemia
Yang's method (2014)	24498199	propagation algorithm on a coding-non-coding gene–disease bipartite network	gene-disease associations	Alzheimer's disease<br>pancreatic cancer<br>gastric cancer
KATZLDA (2015)	26577439	KATZ measure, heterogeneous network	lncRNA-disease associations, lncRNA expression profiles, lncRNA functional similarity, disease semantic similarity, Gaussian interaction profile kernel similarity	colon<br>gastric<br>renal cancer
Liu's method (2014)	24392133	tissue specificity scores calculation, Spearman's correlation coefficients, co-expression	human lncRNA expression profiles, gene expression profiles, human disease-associated gene data	Ovary-related diseases,<br>Testes-related diseases

For each model, we report its name (NAME), its date of publication (DATE), its pubmed id (PUBMED ID), its main method applied in the model (METHOD), its dataset used in the model (DATASETS) and its case studies of diseases (CASE STUDIES).

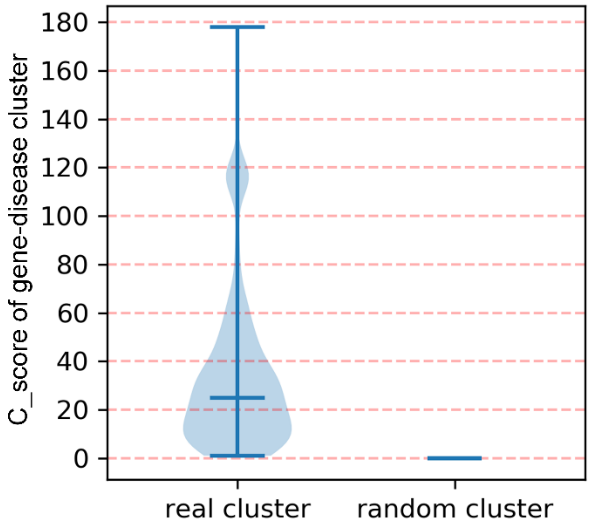

Fig. S1 Comparison of C_score between real gene-disease clusters and random gene-disease cluster.
