# Supplementary material for: Cluster correlation based method for lncRNA-disease association prediction
Source: BMC Bioinformatics. 2020 May 11;21:180. doi: 10.1186/s12859-020-3496-8 (PMC7216352; doi:10.1186/s12859-020-3496-8)
Supplement: Supplementary file 3 — Additional file 3. In this file we provide the details about how to calculate the similarity of gene cluster, the similarity of diseases cluster, the gene-disease association score as well as the procedure of leave-one-out cross validation. [file 12859_2020_3496_MOESM3_ESM.docx]

Supplementary Methods

**1. Calculation of the similarity of gene cluster**

1. For a given disease $d$, we obtain the gene cluster corresponding to $d$.
2. The similarity of any two genes in the gene cluster is calculated by the GOSim[1] package in R. Firstly, we calculated Resnik’s pairwise similarity between GO terms of two genes[2, 3]. Secondly, we calculated similarity of two genes by the average similarity of the GO terms.
3. The similarity of gene cluster is obtained by the average of similarities of any two genes in the gene cluster.
4. For each disease, the similarity of its corresponding gene cluster is calculated by the above process.
5. For gene clusters with same size, we calculate the average of their similarities as the similarity of the gene cluster of that size.
6. For clusters of any size, we generate random gene cluster of the same size and calculated its similarity through the previous process.

**2. Calculation of the similarity of disease cluster**

1. For a given gene $g$, we obtain the disease cluster corresponding to $g$.
2. The similarity of any two diseases in the disease cluster is calculated by the method[4] which was developed by a text-mining approach to map relationships between more than 5000 human genetic disease phenotypes from the OMIM database[5].
3. The similarity of disease cluster is obtained by the average of similarities of any two diseases in the disease cluster.
4. For each gene, the similarity of its corresponding disease cluster is calculated by the above process.
5. For disease clusters with same size, we calculate the average of their similarities as the similarity of the disease cluster of that size.
6. For clusters of any size, we generate random disease cluster of the same size and calculated its similarity through the previous process.

**3. Calculation of disease-gene association scores**

1. For any edge $l$ in the bipartite network which corresponding two nodes, find the disease cluster and gene cluster corresponding to the edge.
2. Calculate the number of connected edges between the two clusters above, which is the $C\_score$ of this edge.
3. Construct a random edge corresponding to $l$. Generate its random gene cluster and random disease cluster whose sizes are same with the gene cluster and the disease cluster of $l$, respectively.
4. Calculate the number of connected edges between the two random clusters above, which is the $C\_score$ of this random edge in an experiment.
5. Repeat the above process 1000 times and calculate the average value as the $C\_score$ for this random edge.

**4. Leave-one-out cross validation based on Yang’s dataset**

1. Data preprocessing. For each edge in the bipartite network, if the size of its disease cluster or lncRNA cluster is less than 2, the edge is deleted. In the end, we kept 236 edges in the bipartite network between 102 diseases and 44 lncRNAs as positive samples.
2. Randomly selected 236 unassociated lncRNA-disease pairs between 102 diseases and 44 lncRNAs as negative samples.
3. Each time select one edge which has not been selected before in the positive samples to delete, then calculate the $DG\_score$ of all edges on the remaining network and get the ranking of the edge deleted and the ranking of edges in the negative samples.
4. Obtain true positive rate (TPR) and false positive rate (FPR) at different levels of threshold
5. Draw the receiver operating characteristics (ROC) curve.

**5. Leave-one-out cross validation based on Lnc2Cancer2.0 dataset**

1. Data preprocessing. For each edge in the bipartite network, if the size of its disease cluster or lncRNA cluster is less than 2, the edge is deleted. In the end, we kept 1541 edges in the bipartite network between 85 diseases and 249 lncRNAs as positive samples.

2. Randomly selected 1541 unassociated lncRNA-disease pairs between 85 diseases and 249 lncRNAs as negative samples.

3. Each time select one edge which has not been selected before in the positive samples to delete, then calculate the $DG\_score$ of all edges on the remaining network and get the ranking of the edge deleted and the ranking of edges in the negative samples.

4. Obtain true positive rate (TPR) and false positive rate (FPR) at different levels of threshold

5. Draw the receiver operating characteristics (ROC) curve.

References

1. 1. Fröhlich H, Speer N, Poustka A, Beißbarth T. GOSim–an R-package for computation of information theoretic GO similarities between terms and gene products. BMC Bioinformatics. 2007;8(1):166.
2. 2. Resnik P. Using information content to evaluate semantic similarity in a taxonomy. In Proceedings of the 14th International Joint Conference on Artificial Intelligence. 1995;1:448-53.
3. 3. Resnik P. Semantic similarity in a taxonomy: An information-based measure and its application to problems of ambiguity in natural language. Journal of artificial intelligence research. 1999;11:95–130.
4. 4. Van Driel MA, Bruggeman J, Vriend G, Brunner HG, Leunissen JA. A text-mining analysis of the human phenome. Eur J Hum Genet. 2006;14(5):535–42.
5. 5. Hamosh A, Scott AF, Amberger JS, Bocchini CA, McKusick VA. Online Mendelian Inheritance in Man (OMIM), a knowledgebase of human genes and genetic disorders. Nucleic Acids Res. 2005;33(suppl_1):D514–7.
